# 99. Immune Responses to Influenza Vaccines in Children and Young Adults

**DOI:** 10.1093/ofid/ofab466.099

**Published:** 2021-12-04

**Authors:** Katherine V Williams, Bo Zhai, John F Alcorn, Mary Patricia Nowalk, Brendan Flannery, Min Levine, Krissy Moehling, Richard K Zimmerman, Judith M Martin

**Affiliations:** 1 University of Pittsburgh, Pittsburgh, Pennsylvania; 2 Centers for Disease Control and Prevention, Atlanta, GA; 3 Centers for Diease Control and Prevention, Atlanta, GA

## Abstract

**Background:**

Immune responses to influenza vaccines (IV) are influenced by pre-existing antibodies to vaccine components. Immune responses to vaccines were evaluated following vaccination with quadrivalent egg-based live-attenuated influenza vaccine (LAIV4) and cell-culture inactivated influenza vaccine (ccIIV4).

**Methods:**

Racially diverse (48.0% non-white), healthy, community-dwelling children and young adults aged 4-21 years (median, 18.3 years) were randomized 1:1 in blocks of 4 to receive intramuscular ccIIV4 (Flucelvax: n=100) or nasal LAIV4 (FluMist: n=98); baseline demographics were similar between groups. Blood was drawn at day 0 pre-vaccination and at day 28 (21-35 days) post vaccination. Hemagglutination inhibition (HI) assays against egg-grown A/H1N1, A/H3N2, both vaccine B/strains and cell-grown A/H3N2 antigens were conducted. Geometric mean titers (GMT) and geometric mean fold rise (GMFR) in titers were analyzed.

**Results:**

Day 0 GMTs were similar for LAIV4 and ccIIV4. Day 28 GMTs were higher for ccIIV4 (p< 0.05) and increased following vaccination for all 5 antigens (p< 0.05) except B/Phuket following LAIV4. The GMFR range was 2.4 to 3.0 for ccIIV4 and 1.0 to 1.3 for LAIV4. In linear regression controlling for age and prior season vaccination for both vaccines, baseline titers inversely predicted GMFR. The GMFR to A/H3N2 cell-grown and egg-grown antigens were similar within vaccine type.

Figure 1.

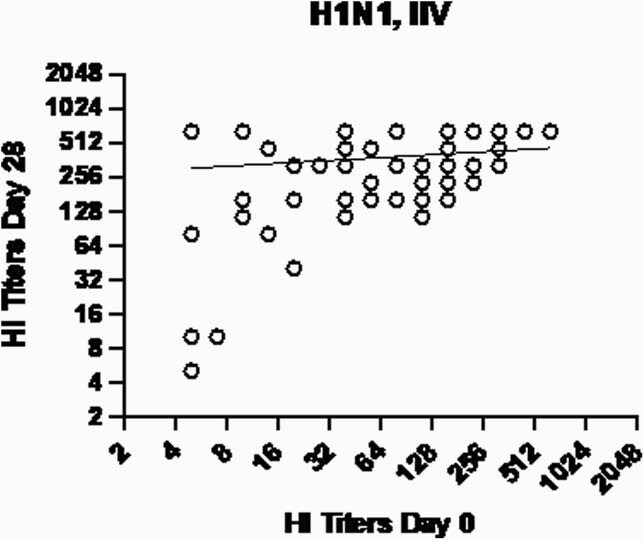

Day 0 and Day 28 A(H1N1) titers for ccIIV4

Figure 2.

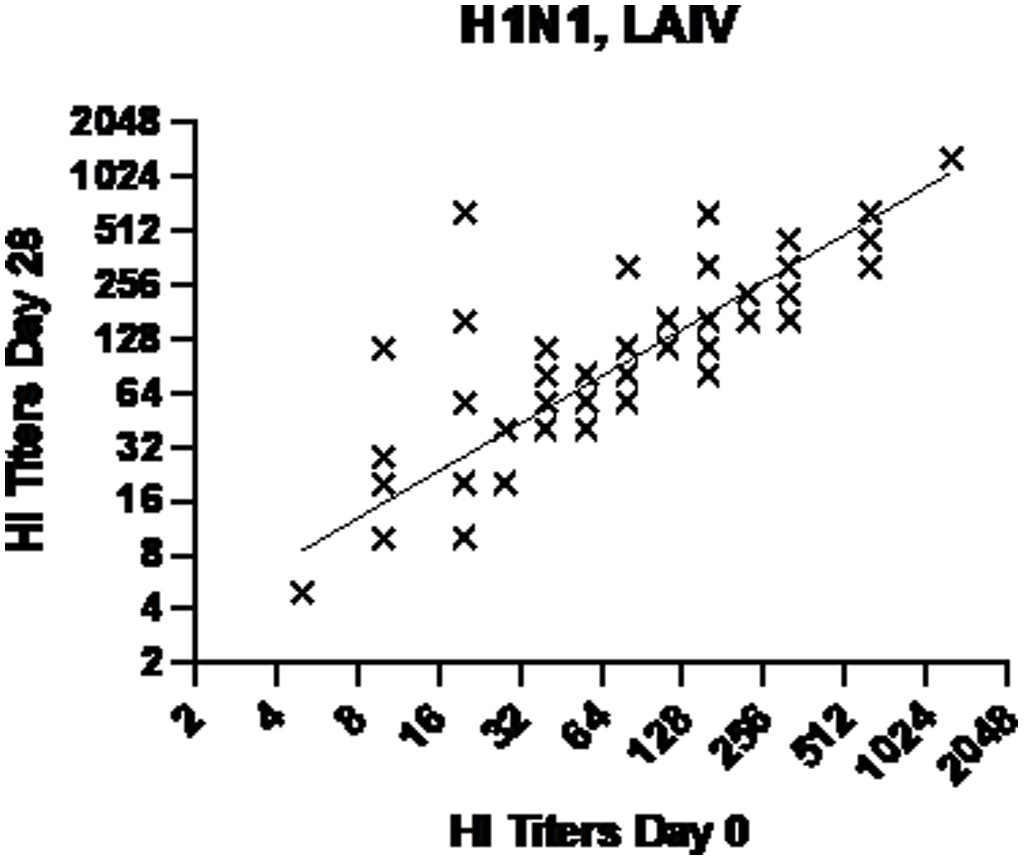

Day 0 and Day 28 A(H1N1) titers for LAIV4

**Conclusion:**

The HI response to ccIIV4 was greater than LAIV4 in this study of mostly older children. Day 0 HI titers were 1) a significant determinant of GMFR; 2) the strongest predictors of day 28 GMFR; and 3) more highly correlated (negatively) with GMFR following ccIIV4 than LAIV4. For both IV, the GMFR for cell-grown and egg-grown A/H3N2 antigens did not differ within IV type. Future studies incorporating immunoglobulin and cellular immune responses may delineate differences between these IV types not observable through HI assays.

**Disclosures:**

**Mary Patricia Nowalk, PhD**, **Merck & Co., Inc.** (Grant/Research Support) **Richard K. Zimmerman, MA;MD;MPH;MS**, **Sanofi Pasteur** (Research Grant or Support) **Judith M. Martin, MD**, **Merck Sharp and Dohme** (Consultant)

